# Granulosa cell tumor patients presenting with acute abdomen: a case series

**DOI:** 10.1016/j.gore.2025.101781

**Published:** 2025-06-13

**Authors:** Geertruid J. Brink, Jolijn W. Groeneweg, Ariane A. Sickinghe, Christianne A.R. Lok, Hans W. Nijman, Jurgen M.J. Piek, Ward Hofhuis, Eva Maria Roes, Luc R.C.W. van Lonkhuijzen, Cor D. de Kroon, Eelke H. Gort, Petronella O. Witteveen, Ronald P. Zweemer

**Affiliations:** a Department of Gynecologic Oncology, University Medical Center Utrecht, Utrecht, the Netherlands; b Department of Gynecological Oncology, Center for Gynecological Oncology Amsterdam, Antoni van Leeuwenhoek Hospital, Amsterdam, the Netherlands; c Department of Obstetrics and Gynecology, University Medical Center Groningen, Groningen, the Netherlands; d Department of Obstetrics and Gynecology, and Catharina Cancer Institute, Catharina Hospital, Eindhoven, the Netherlands; e Department of Obstetrics and Gynecology, Franciscus Gasthuis en Vlietland, Rotterdam, the Netherlands; f Department of Gynecologic Oncology, Erasmus MC Cancer Institute, Rotterdam, the Netherlands; g Center for Gynecological Oncology, Cancer Center Amsterdam, Amsterdam University Medical Center, Amsterdam, the Netherlands; h Department of Obstetrics and Gynecology, Leiden University Medical Center, Leiden, the Netherlands; i Department of Medical Oncology, University Medical Center Utrecht, Utrecht, the Netherlands

**Keywords:** Acute abdomen, Differential diagnosis, Granulosa cell tumor, Hemoperitoneum, Ovarian cancer, Ovarian mass, Presentation, Rare disease

## Abstract

**Objective:**

To highlight the unique clinical presentation of acute abdomen in granulosa cell tumor patients.

**Methods:**

In a multicenter cohort study of adult-type granulosa cell tumors, women presenting with an acute abdomen at diagnosis were identified (Brink, 2025). After informed consent, clinical data were retrieved from patient records and collected in a Castor EDC database. Statistical analyses were performed to compare the subgroup of women with acute abdomen with the remainder of the adult type granulosa cell tumor cohort.

**Results:**

Twenty-six out of 208 (12.5%) patients had an acute abdomen as presenting symptom of an adult-type granulosa cell tumor. All patients underwent emergency abdominal surgery. In 16 (62%) patients, the tumor mass was found to be ruptured pre-operatively, and 7 (27%) patients had an ovarian torsion. Seventeen patients (65%) of this group developed recurrent disease. When compared with the rest of the adult-type granulosa cell tumor cohort, patients with an acute abdomen at diagnosis were younger, had significantly more perioperative blood loss, and more often developed a recurrence.

**Conclusions:**

Adult-type granulosa cell tumors present with an acute abdomen in over 10% of the cases. In case of an ovarian mass or hemoperitoneum in women with an acute abdomen, a granulosa cell tumor should be considered in the differential diagnosis. Surgery can then be performed with a more oncological approach, striving to avoid spill and thus decrease the risk of recurrence.

## Highlights

• Acute abdomen was the first symptom in 12.5% of adult-type granulosa cell tumor patients.
• Most patients with acute abdomen had a ruptured tumor or ovarian torsion requiring emergency surgery.
• Granulosa cell tumor patients with acute abdomen were younger and had higher perioperative blood loss than others.
• Awareness of granulosa cell tumor as a differential diagnosis in acute abdomen may improve surgical outcomes.

## Graphical abstract

**Figure:**
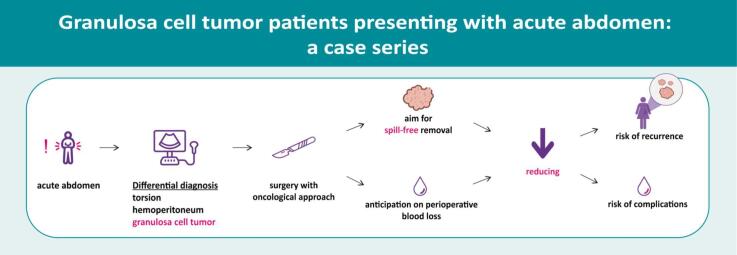

## 1 Introduction

Adult-type granulosa cell tumor (aGCT) originates from ovarian granulosa cells. Granulosa cells are a hormonally active component of the ovarian stroma, responsible for estrogen production. Uncontrolled growth of these granulosa cells can lead to the formation of a granulosa cell tumor, an ovarian cancer subtype representing 3–5 % of ovarian malignancies (Schumer and Cannistra, 2003, Ferlay et al., 2024). Due to the production of estrogen, vaginal bleeding can be the first symptom of an aGCT and concurrent endometrial hyperplasia or carcinoma is found in a subset of patients (Schumer and Cannistra, 2003). Other possible symptoms of aGCT include abdominal pain or gastrointestinal complaints. Sometimes aGCT is asymptomatic and diagnosed after a coincidental finding on ultrasound or other abdominal imaging. The presentation of aGCT differs from patients with epithelial ovarian cancer, who typically present at an advanced stage, with abdominal discomfort, distention or bloating, or loss of appetite (Ebell et al., 2016).In literature, a subset of patients is reported in whom an acute abdomen was the first presentation of an aGCT, requiring emergency surgical treatment (Brink, 2025).

Several case reports also describe aGCT to present with an acute abdomen, defined as a sudden onset of severe abdominal pain, typically requiring urgent surgical evaluation. Acute abdomen can be caused either by torsion of the ovarian mass (Siviero, 2020, Zara and Gilardi, 1968, Zhang et al., 2022) or by rupture of the aGCT, leading to a hemoperitoneum (Anbarasu et al., 2020, Aydin et al., 2001, Bastu, 2013, Bedir, 2014, Borah et al., 2010, Givalos, 2005, Habek et al., 2003, Kakani et al., 2012, Lee et al., 1999, Oge, 2012, Poma, 1998). Hemoperitoneum can lead to acute abdomen by causing peritoneal irritation and increased intra-abdominal pressure due to the accumulation of blood within the peritoneal cavity, resulting in severe abdominal pain. The removal of the adnexal mass in the acute setting carries a higher risk of spill, which is a known risk factor for a worse prognosis (Brink, 2025, Plett, 2023). No previous case series has described this presenting symptom of aGCT.

With this case series, we aim to highlight the unique clinical presentation of a subset of aGCT patients presenting with acute abdomen.

## 2 Methods

### 2.1 Study population

All aGCT patients participating in the GRANULOSA study who had initially presented with acute abdomen were identified. The GRANULOSA study is an ongoing nationwide prospective cohort study in the Netherlands. Patients suspected of or diagnosed with a granulosa cell tumor, both juvenile and adult type, are recruited for the study. No juvenile granulosa cell tumor patients were included in this analysis. All participating patients gave written informed consent for their data to be used for study purposes, the study has been approved by the medical ethical committee of the UMCU (METC 17–868). From April 2018 to April 2024, patients were enrolled at the time of diagnosis of aGCT, or at any other time in the course of the disease or during follow-up.

### 2.2 Outcomes

For the GRANULOSA study, the following clinical data were collected: baseline characteristics, age at diagnosis, symptoms at diagnosis, FIGO stage at diagnosis (Prat, 2014), treatment, number of recurrences and disease status. In addition, the medical files were screened for the occurrence of an acute abdomen at first presentation. Acute abdomen was defined as a sudden onset of severe abdominal pain, leading to an urgent medical intervention. For the patients presenting with acute abdomen, the following additional parameters were collected: days between symptoms and presentation at hospital, days between presentation at hospital and surgery, and the presence of ovarian torsion. Disease status at the end of follow-up was reported in four categories: no evidence of disease (NED), alive with disease (AWD), death of disease (DOD) or death of other cause (DOC).

### 2.3 Data collection

Data were retrieved from participating hospitals in the Netherlands. Clinical data were derived from electronic health records, pseudonymized and stored in the Castor EDC database (Castor, 2019). If the medical records were incomplete, records were requested from local gynecologists and hospitals. Data collection started in April 2018 and patients were followed-up until April 2024.

### 2.4 Statistical analysis

Statistical analyses were performed using SPSS Statistics for Windows, Version 29.0.1 (IBM Corp., Armonk, NY, USA). Continuous variables are presented as median with their range and categorical variables as number and percentage of the total.

For the comparisons between the group presenting with an acute abdomen and the non-acute presentation group, the Mann-Whitney *U* test was used for continuous variables and the Chi-square test for categorical variables. A Kaplan-Meier survival analysis was conducted to compare the survival distributions between the two groups, with the log-rank test used to assess the statistical significance of the differences between the survival curves. The time axis for the Kaplan-Meier curve of overall survival was limited to 30 years, and for recurrence-free survival, it was limited to 15 years.

## 3 Results

In our cohort of 208 histologically confirmed aGCT patients 26 patients (12.5 %) were identified with an acute abdomen. The other 182 patients presented mostly with abdominal pain (19.2 %), vaginal bleeding (33.5 %) or both (10.4 %). Clinical parameters of the patients presenting with an acute abdomen are summarized in Table 1. Median duration between the start of complaints and presentation at the hospital was one day (range 0 – 21 days). Median duration between presentation of the patient at the hospital and surgery was zero days (range 0 – 21 days). Three patients presented with an acute abdomen, however the symptoms resolved spontaneously allowing for surgery in a planned setting.

**Table 1:** Clinical summary of 26 adult-type granulosa cell tumor patients presenting with acute abdomen.

**Patient**	**Age at diagnosis (y)**	**Days between presentation at hospital and operation**	**Type of surgery**	**Conversion during surgery**	**Mass status**	**Torsion of ovary**	**Blood lossin ml**	**Surgery second tempi**	**Clinical FIGO stage at diagnosis**	**FU/RFS since diagnosis** **in years**	**Number of recurrences after diagnosis**	**Disease status**
1	35	7	SO	−		−	10	+	IIB^a^	4.57	−	NED
2	43	0	SO	−		−	200	−	IC	8.50	−	NED
3	40	0	SO	−		+	2400	−	IC	3.68	−	NED
4	61	21	TAH + BSO + A	−		−	100	−	IA	0.13	−	NED
5	36	0	O	+		−	2300	−	IC	3.08	−	NED
6	52	0	SO	−		+	500	−	IA	7.72	−	NED
7	52	12	SO	−		−	10	−	IA	2.07	−	NED
8	34	9	SO	−		+	50	−	IA	2.64	−	NED
9	58	0	BSO	−		−	2500	−	IC	0.30	−	NED
10	45	0	SO	−		+	1050	−	IC	5.27	4	AWD
11	36	0	SO	−		U	400	−	IC	5.62	2	AWD
12	30	0	SO	−		−	600	−	IC	7.05	1	NED
13	62	1	BSO	−		−	U	+	IIB	6.38	1	NED
14	59	0	SO	−		+	600	−	IC	2.60	3	AWD
15	48	0	SO	−		−	50	−	IC	3.09	1	NED
16	44	0	O	−		+	300	−	IC	2.13	3	AWD
17	45	0	SO	−		−	U	−	IC	8.32	7	AWD
18	60	0	BSO^b^	−		−	1000	−	IC	4.17	3	NED
19	50	0	DLC + B^b^	−		−	U	+	IC	3.99	3	AWD
20	48	3	BSO^b^	−		−	3600	−	IC	18.38	2	AWD
21	37	0	SO	−		−	100	−	IA	3.96	1	NED
22	39	0	SO	+		−	2000	−	IA	3.57	3	AWD
23	52	1	SO	−		−	300	−	IIA	3.52	3	AWD
24	49	0	SO	+		−	200	−	IC	19.88	4	DOD
25	44	0	SO	+		−	1100	−	IC	1.15	5	DOD
26	53	0	SO	−		+	10	−	IC	1.06	1	DOD

A: appendectomy, AWD: alive with disease, B: biopsy, BSO: bilateral salpingo-oophorectomy, DLC: double loop colostomy, DOD: dead of disease, FU: follow-up, NED: no evidence of disease, O: oophorectomy unilateral, RFS: recurrence free survival, SO: salpingo-oophorectomy unilateral, TAH: total abdominal hysterectomy, U: unknown, : encapsulated, : surgical spill, : ruptured.

a Surgically staged. ^b^ P18, 19,20 received adjuvant chemotherapy.

In four cases (15.4 %) the surgery started laparoscopically but was converted to laparotomy. In 16 cases (61.5 %), the tumor had already ruptured. In seven cases (26.9 %) a torsion of the ovarian tumor was observed. Median blood loss was 500 ml (10 – 3600 ml). In three patients, a second surgery followed for either staging or additional debulking surgery. Three patients (11.5 %) were treated with adjuvant chemotherapy (carboplatin/etoposide, cisplatin/etoposide, or bleomycin/etoposide/cisplatin). No other systemic treatments were administered at the time of the first diagnosis. In most cases, emergency surgery was performed by general gynecologists, typically in non-academic centers. One patient was surgically staged (peritoneal staging only) and had FIGO stage IIB disease. The remaining 25 patients were not surgically staged and their FIGO stage was determined based on clinical and imaging data. Six patients (23.1 %) had FIGO stage IA, 17 patients (65.4 %) FIGO stage IC, one patient (3.8 %) FIGO stage IIA and one patient (3.8 %) FIGO stage IIB disease.

Median follow-up of the 26 patients was 8.15 years (range 1.51 months – 32.58 years). At the time of the last follow-up, nine patients (34.6 %) were disease-free. Seventeen patients (65.4 %) developed a recurrence with a median recurrence free interval of 3.99 years (range 1.06 – 19.88 years). Recurrence occurred in 75 % of patients with a ruptured tumor versus 33 % of those with an intact capsule, although this difference was not statistically significant (p = 0.138).

At the end of follow-up 14 patients (53.8 %) had no evidence of disease, nine patients (34.6 %) were alive with disease and three patients (11.5 %) had died of an aGCT.

**Fig. 1:**
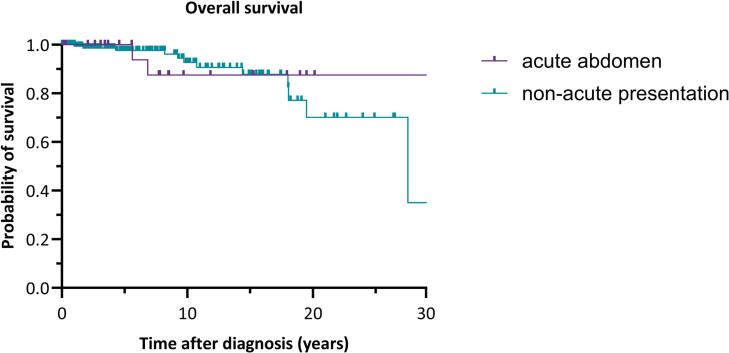
Kaplan Meier curve for the overall survival over 30 years of adult-type granulosa cell tumor patients with acute abdomen and those with a non-acute presentation. Log-rank test: χ^2^(1) = 0.0838, p = 0.772.

**Fig. 2:**
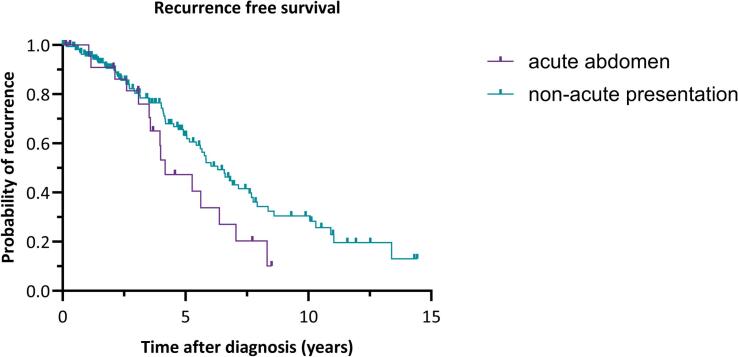
Kaplan Meier curve for the recurrence free survival over 15 years of aGCT patients with acute abdomen and those with a non-acute presentation. Log-rank test: χ^2^(1) = 3.260, p = 0.071.

A comparison of several baseline characteristics between aGCT patients presenting with acute abdomen (n = 26) and non-acute presentation (n = 182) was made, as shown in Table 2. The acute abdomen group was significantly younger than the non-acute group, z = -2.86, p = 0.004. The amount of perioperative blood loss was significantly higher in the group with an acute abdomen, z = -2.023, p = 0.043. A recurrence occurred significantly more often in the acute abdomen group (p = 0.034). No significant differences were observed in BMI, type of secondary surgery, tumor size, and disease status. In addition, no significant differences in overall survival or recurrence free survival between the two groups were found, as shown in Fig. 1, Fig. 2.

**Table 2:** Comparison of baseline characteristics between adult-type granulosa cell tumor patients with acute abdomen and patients with a non-acute presentation.

**Characteristic**		**Acute abdomen** n (%) median (min – max)	**Non-acute presentation** n (%) median (min – max)	**p-value**
Number of patients		26 (100)	182 (100)	

Age at diagnosis in years		46.5 (30 – 62)	56.0 (20 – 84)	0.004

BMI at inclusion in kg/m^2^		27.3 (21.0 – 42.0)	25.8 (18.0 – 60.2)	

Initial surgery				<0.001

	Cystectomy unilateral	0 (0)	11 (6.0)	

	Ovariectomy unilateral	2 (7.7)	5 (2.7)	

	Adnexectomy unilateral	18 (69.2)	46 (25.3)	

	BSO	4 (15.4)	27 (14.8)	

	BSO + hysterectomy	1 (3.8)	50 (27.5)	

	Complete peritoneal staging	0 (0)	29 (15.9)	

	Other	1 (3.8)	14 (7.7)	

Perioperative blood loss in mL		500 (10 – 3600)	200 (10 – 4000)	0.043

Secondary surgery				

	Complete staging surgery	1 (3.8)	16 (8.8)	

	Incomplete staging surgery	2 (7.7)	13 (7.1)	

	No secondary surgery	23 (88.5)	153 (84.1)	

Adjuvant treatment				0.039

	Chemotherapy	3 (11.5)	4 (2.2)	

	Radiotherapy	0 (0)	3 (1.6)	

	No adjuvant treatment	23 (88.4)	175 (96.2)	

Tumor capsule				<0.001

	Capsule intact	6 (23.1)	98 (53.8)	

	Ruptured	16 (61.5)	21 (11.5)	

	Surgical spill	4 (15.4)	48 (26.4)	

	Unknown	0 (0)	15 (8.2)	

Tumor size in cm		11.0 (1.9 – 25.0)	10.0 (0.8 – 36.0)	

Recurrence after primary diagnosis		17 (65.4)	76 (41.8)	0.034

Disease status				

	No evidence of disease	14 (53.8)	134 (73.6)	

	Alive with disease	9 (34.6)	35 (19.2)	

	Death of disease	3 (11.5)	12 (6.6)	

	Death of other cause	0 (0)	1 (0.5)	

BMI: body mass index, BSO: bilateral salpingo-oophorectomy, cm: centimeters, min: minimum, max: maximum.

## 4 Discussion

### 4.1 Main findings

In our cohort of 208 aGCT patients, 26 patients (12.5 %) presented with acute abdominal pain. In more than 60 % of the patients the tumor was found to be ruptured upon surgical opening of the abdomen and in more than 25 % there was an ovarian torsion. Patients presenting with acute abdominal pain were significantly younger, had increased perioperative blood loss, and developed a recurrence more often, as compared to the non-acute group.

### 4.2 Interpretation

When female patients with an acute abdomen are assessed at the emergency department, the differential diagnosis according to Cartwright and Knudson (Cartwright and Knudson, 2008), consists of appendicitis, colitis, diverticulitis, inflammatory bowel disease, ectopic pregnancy, fibroids, ovarian mass, torsion, pelvic inflammatory disease, nephrolithiasis and pyelonephritis (Cartwright and Knudson, 2008, McWilliams et al., 2008). Presentation of acute abdomen reported in cancer cases is mostly described when the diagnosis is known, e.g. following treatment. Current literature harbors a few reports of acute abdomen as first presentation of a malignancy in colon cancer and ovarian cancer (Ilgen and Marr, 2009). Ovarian cancer is known as the ‘silent killer’ in part because symptoms are absent or not rapidly progressive changing so slowly that patients do not feel alarmed (Jayson et al., 2014, Lheureux et al., 2019). Although case reports have previously shown that aGCT patients can present with an acute abdomen due to hemoperitoneum or torsion, the typical presentation of aGCT is non-acute abdominal pain with or without vaginal bleeding (Brink, 2025). The presentation of ovarian cancer patients with an acute abdomen at diagnosis is considered to be rare. However, this case series represents a unique and relatively large subset of aGCT with an acute abdomen at initial presentation.

If gynecologists are attentive to the possibility of aGCT in case of an acute abdomen and ovarian mass, surgery and further peritoneal inspection can be performed with an oncological approach. in cases where the tumor is still intact, spill-free removal should be aimed for. As shown in previous studies, capsule rupture is a risk factor for the development of recurrence (Plett, 2023, Wilson, 2015). Therefore, it is important to consider aGCT as part of the differential diagnosis of an acute abdomen.

### 4.3 Clinical and research implications

With this study, we aimed to highlight the unique clinical presentation of a subset of aGCT patients presenting with an acute abdomen. Although emergency surgery cannot be avoided in these cases, early recognition of aGCT prior to surgery is important to optimize surgical management. Awareness of the potential diagnosis could lead to surgery with a more oncologic approach, minimizing the risk of intraoperative tumor rupture and peritoneal spill where feasible, which may reduce the risk of recurrence. Early recognition may also allow for peritoneal staging during the initial procedure, which is often omitted in emergency settings but can provide valuable prognostic information. Furthermore, anticipating significant intraoperative blood loss in these patients enables better perioperative preparation and resource allocation. These findings highlight the need to increase awareness of aGCT as a possible underlying cause of acute abdomen in women.

Future research should focus on improving the preoperative identification of aGCT in patients presenting with acute abdominal symptoms. Studies evaluating characteristic features of aGCT on imaging modalities, including ultrasound, CT, and MRI, could enhance diagnostic accuracy. Additionally, exploring the biological mechanisms underlying the development of aGCT could provide valuable insights for early detection. Exploring genetic factors, such as the FOXL2 mutation, may help in understanding the disease's onset and progression, ultimately aiding in diagnostic strategies for patients with suspected aGCT.

### 4.4 Strengths and limitations

The main strength of this study is the aggregation of clinical data from a subgroup of aGCT patients with an atypical presentation, providing a clearer overview of this subgroup. While separate case reports have been published previously, this is the first time that clinical data from these patients has been collectively analyzed and compared with the cohort from which they were drawn.

A limitation of this study is the combination of both retrospective and prospective collection of data, which plausibly caused selection bias. A significant portion of the patients in our original cohort was retrospectively included, with a longer median follow-up duration, which likely contributed to a higher observed incidence of recurrences.. Conducting a purely prospective study on this rare tumor would be challenging and would require a follow-up period of over ten years, considering the potential of aGCT to recur after numerous years.

## 5 Conclusion

This case series highlights the high incidence of patients with an aGCT presenting with an acute abdomen. Preoperative recognition of the possibility of aGCT and using an oncologic approach during surgery, could improve long-term outcomes. Therefore, we urge that in the differential diagnosis of acute abdomen with an ovarian mass or hemoperitoneum in a middle-aged woman, a granulosa cell tumor should be considered.

## CRediT authorship contribution statement

**Geertruid J. Brink:** Writing – review & editing, Writing – original draft, Visualization, Project administration, Formal analysis, Conceptualization. **Jolijn W. Groeneweg:** Writing – review & editing, Writing – original draft, Validation, Supervision, Formal analysis, Conceptualization. **Ariane A. Sickinghe:** Writing – review & editing, Investigation. **Christianne A.R. Lok:** Writing – review & editing, Resources. **Hans W. Nijman:** Writing – review & editing, Resources. **Jurgen M.J. Piek:** Writing – review & editing, Resources. **Ward Hofhuis:** Writing – review & editing, Resources. **Eva Maria Roes:** Writing – review & editing, Resources. **Luc R.C.W. van Lonkhuijzen:** Writing – review & editing, Resources. **Cor D. de Kroon:** Writing – review & editing, Resources. **Eelke H. Gort:** Writing – review & editing, Supervision. **Petronella O. Witteveen:** Writing – review & editing, Validation, Supervision, Conceptualization. **Ronald P. Zweemer:** Writing – review & editing, Validation, Supervision, Methodology, Conceptualization.

## Declaration of competing interest

The authors declare that they have no known competing financial interests or personal relationships that could have appeared to influence the work reported in this paper.
